# Intratracheal inoculation of human varicella zoster virus (VZV; MAV strain) vaccine successfully induced VZV IgG antibodies in rhesus monkeys

**DOI:** 10.1186/s42826-021-00091-3

**Published:** 2021-05-22

**Authors:** Jong-Min Kim, Chung-Gyu Park

**Affiliations:** 1 Xenotransplantation Research Center, Seoul National University Graduate School, Seoul, Korea; 2 Institute of Endemic Diseases, Seoul National University Graduate School, Seoul, Korea; 3 Cancer Research Institute, Seoul National University Graduate School, Seoul, Korea; 4 Biomedical Research Institute, Seoul National University Hospital, Seoul, 110-799 Korea; 5 Department of Microbiology and Immunology, Seoul National University Graduate School, Seoul, Korea; 6 Department of Biomedical Sciences, Seoul National University Graduate School, Seoul, Korea; 7 Department of Microbiology and Immunology, Department of Biomedical Sciences, Xenotransplantation Research Center, Cancer Research Institute, Seoul National University College of Medicine, 103 Daehak-ro Jongno-gu, Seoul, 110-799 Korea

**Keywords:** Simian varicella virus, Varicella-zoster virus, Immunization, Latent infection, Rhesus monkey

## Abstract

**Background:**

The objective of this study was to investigate whether the use of live attenuated varicella zoster virus (VZV) MAV vaccination can efficiently induce VZV antibody production in naive rhesus monkeys as an approach to prevent simian varicella virus (SVV) reactivation in animals immunosuppressed for transplantation studies.

**Results:**

Clinically available human VZV vaccine was used to induce the production of anti-VZV antibodies in rhesus monkeys. A vial of the vaccine was subcutaneously injected at 0 week, and the second and third vaccination was performed at 5 and 6 weeks by intratracheal inoculation. The titer of anti-VZV IgG was assessed at 0, 2, 4, 6, and 7 weeks. At 2 weeks, 3/16 were seropositive for VZV IgG. At 6 weeks, 9/16 were shown to be seropositive. At 7 weeks, 16/16 were found to be seropositive.

**Conclusions:**

The VZV vaccine via intratrachael inoculation was shown to induce VZV IgG humoral immunity in rhesus monkeys and may be important immunosuppressed macaques for transplantation studies. Although the humoral immunity produced is an important finding, further studies will be necessary to confirm possible protection and it could protect probably against SVV infection in rhesus monkey.

## Background

Simian varicella virus (SVV) in Old World monkeys causes epizootic erythematous disease, which is characterized by fever, vesicular skin rash, hepatitis, and high morbidity and mortality [1]. Outbreaks of SVV occur sporadically in facilities housing nonhuman primates and can result in a loss of valuable research animals [1]. When SVV infection outbreaks occur, prompt diagnosis and antiviral treatment are important for patients at an early time point. In nonhuman primate (NHP) transplantation research involving the use of immunosuppressive drugs or in NHPs under immunocompromised status, it is important to prevent the recurrence of latent viruses in hosts that do not respond normally to infection following immunosuppression that produces a weakened immune system. Human varicella-zoster virus (VZV) infection shows clinical, pathological, and virologic features similar to those of SVV infection [2]. VZV infection, which does not induce clinical symptoms in macaques, might be useful to protect monkeys against SVV disease [3]. Thus, the objective of this study was to investigate whether the use of live attenuated VZV MAV vaccination can efficiently induce VZV antibody production in naive rhesus monkeys.

## Results

### Background history

**Fig. 1:**
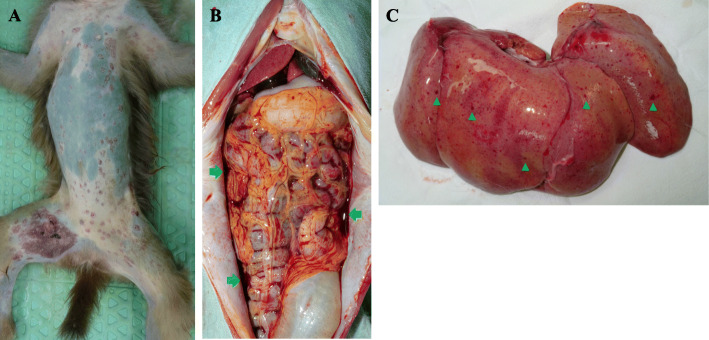
Images from necropsy on SVV-infected rhesus monkey. **a** Vesicular skin rash is shown on skin. **b** Hemoperitonium (arrow) due to hemorrhage is observed in this severely infected monkey. **c** hepatic petechiae (arrowhead) by hemorrhage is observed

R1 (ID of rhesus monkey number 1), an immunosuppressed rhesus monkey, for porcine islet transplantation among 14 rhesus monkeys (8 naïve, 6 immunosuppressed), housed in Nonhuman Primate Research Center at Seoul National University Hospital exhibited clinical symptoms, such as systemic vesicular rash, fever, anorexia, and vomiting (Table 1). SVV was suspected because of the vesicular skin rash, and SVV viremia was confirmed by qPCR. Anti-herpetic agent (ganciclovir 5 mg/kg, IV, once a day), antibiotics (cefazolin 30 mg/kg, IV, twice a day), NSAIDs (ibuprofen 30 mg/kg, PO, in case of necessity), TNF α blocker (adalimumab, 5 mg/kg, SC, once), and antiemetic (maropitant citrate, 1 mg/kg, SC, once a day) were administered to treat SVV and to alleviate symptoms. Two days after the onset of symptoms, serum AST and ALT levels were raised 10-fold relative to the upper limit (AST: 46.7, ALT: 63.5) of normal [4]. The monkey died 3 days after the onset of symptoms. Hemoperitoneum and hepatic petechiae were observed on necropsy (Fig. 1). The day after R1 displayed clinical symptoms, R2, R3, and R4 developed systemic vesicular skin rashes, and SVV viremia was also detected in these monkeys. Ganciclovir and cefazolin, +/− ibuprofen +/− antiemetic were administered to treat SVV and to alleviate symptoms. R2, R3, and R4 survived after receiving these treatments. The day after R1 displayed clinical symptoms, the asymptomatic monkeys received prophylactic ganciclovir. One week after R1 became symptomatic, the asymptomatic monkeys underwent qPCR testing for SVV, which confirmed SVV viremia in R5 and R6; these monkeys, however, remained asymptomatic. The absence of symptoms might have resulted from the administration of prophylactic ganciclovir.

**Table 1:** Summary of simian varicella virus outbreak and follow-up

	Symptoms	SVV viremia (PCR)	Immune status during SVV outbreak	Treatment of SVV	Follow up
R1	Fever Vesicular skin rash Anorexia Vomiting	+	Immunosuppression for islet TPL	Ganciclovir Cefazolin Ibuprofen Adalimumab Maropitant citrate	↑AST and ALT Death 3 days after the onset of symptoms
R2	Fever Vesicular skin rash Anorexia	+	Immunosuppression for islet TPL	Ganciclovir Cefazolin Ibuprofen Adalimumab	Disappearance of rash 5 days after the onset of symptoms Recovery
R3	Fever Vesicular skin rash	+	Immunocompetence	Ganciclovir IV SID Cefazolin Ibuprofen	Disappearance of rash 6 days after the onset of symptoms Recovery
R4	Vesicular skin rash Vomiting	+	Immunocompetence	Ganciclovir IV SID Cefazolin Maropitant citrate	Disappearance of rash 6 days after the onset of symptoms Recovery
R5	–	+	Immunosuppression for islet TPL	Prophylactic use of ganciclovir	SVV viremia detected but no symptoms by prophylactic use of ganciclovir
R6	–	+	Immunocompetence	Prophylactic use of ganciclovir	SVV viremia detected but no symptoms by prophylactic use of ganciclovir
R7	–	–	Immunosuppression for islet TPL	Prophylactic use of ganciclovir	
R8	–	–	Immunocompetence	Prophylactic use of ganciclovir	
R9	–	–	Immunosuppression for islet TPL	Prophylactic use of ganciclovir	
R10	–	–	Immunosuppression for islet TPL	Prophylactic use of ganciclovir	
R11	–	–	Immunocompetence	Prophylactic use of ganciclovir	
R12	–	–	Immunocompetence	Prophylactic use of ganciclovir	
R13	–	–	Immunocompetence	Prophylactic use of ganciclovir	
R14	–	–	Immunocompetence	Prophylactic use of ganciclovir

There were no complications related to subcutaneous or intratracheal injection, such as pain, swelling, and redness at the injection site and fever, irritability, drowsiness, and rash by systemic reaction.

**Fig. 2:**
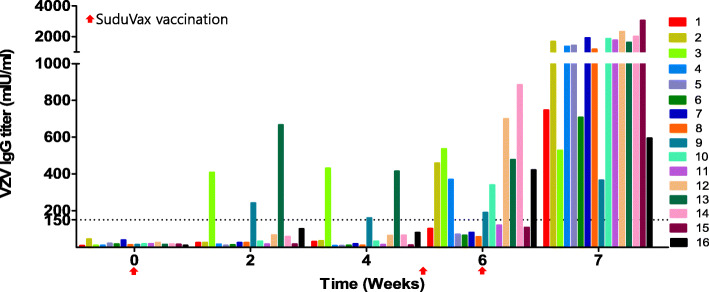
VZA IgG monitoring data. The vaccine was subcutaneously injected at week 0 and administered by intratracheal inoculation at weeks 5 and 6. The dotted line indicates a positive cutoff value

After subcutaneous injection of the vaccine, 3 out of 16 monkeys (18.7%) were seropositive at 2 weeks. At 6 weeks (1 week after the first intratracheal inoculation), 9 out of 16 monkeys (56.2%) were shown to be seropositive. At 7 weeks (1 week after the second intratracheal inoculation), 16 out of 16 monkeys (100%) were found to be seropositive (Fig. 2).

**Fig. 3:**
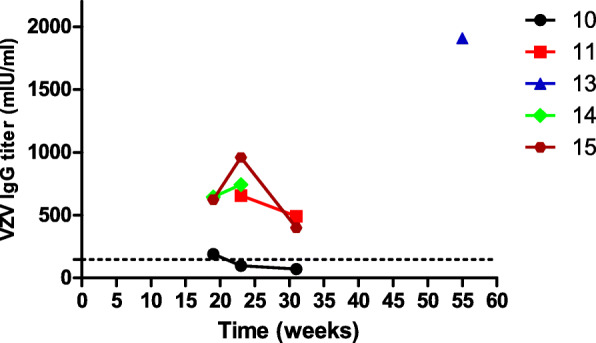
Long-term monitoring of VZV IgG titers in five monkeys. Four out of five rhesus monkeys had VZV IgG titers above cutoff value for at least 31 weeks

Longitudinal data of VZV IgG showed that four out of five monkeys for which serum was available had VZV IgG titers above cutoff value until after about 31 weeks, especially R13, which had high VZV IgG titers until 55 weeks (Fig. 3).

## Discussion

We found that SuduVax (VZV MAV strain), especially via intratracheal inoculation, was able to induce VZV IgG production. Meyer et al. demonstrated a lack of varicella and viral replication in either the lungs or whole blood by wild type VZV intratracheal inoculation in rhesus monkeys. All four rhesus monkeys generated an immune response characterized by the generation of VZV-specific antibodies and T-cells in their study. VZV-immune rhesus monkeys displayed no varicella rash and had lower SVV viral loads as well as earlier and stronger humoral and cellular immune responses than controls in the SVV challenge [3]. Our data showed VZV vaccination of monkeys generated a strong immune response with VZV IgG induction suggesting it may provide protection against latent SVV reactivation and disease. Confirmation of protection, however, would require further testing to prove if the humoral immunity produced is cross-protective, and if vaccination also induces VZV cellular immunity and a high level of protection against SVV challenge. If clinically available, the live attenuated form of the human VZV vaccine might may be useful for immunizing rhesus monkeys and potentially providing protection against SVV disease. One strength of this study is the easy availability of inoculation agent because VZV vaccine is clinically available, but wild-type VZV is not.

The seropositivity of SuduVax (VZV MAV vaccine) by subcutaneous inoculation in humans was shown to be 51 ~ 69% in 1- to 6-year-olds [5]. Since SuduVax is administered subcutaneously in humans, it was also administered subcutaneously in rhesus monkeys. After subcutaneous administration of SuduVax to rhesus monkeys, 18.7% seropositivity was observed in this study, which was relatively low compared to that of humans. A review of the literature confirmed that there was seropositivity in monkeys when intratracheal inoculation of VZV was performed [3], and the inoculation route of SuduVax was changed to intratracheal inoculation. All subjects were seropositive after two intratracheal inoculations.

There were a few limitations of this study. We were not able to did not confirm that VZV IgG could neutralize the SVV antigen due to the absence of the SVV antigen. The SVV IgG level could not be measured because an ELISA kit, which measures only SVV, was not available. A challenge test of SVV in VZV-vaccinated rhesus monkeys was not performed due to lack of pathogenic SVV. In the future, SVV can be obtained from leaders in the SVV field for the needed challenge studies. However, because various studies, especially Meyer et al.’s report [3], have confirmed cross-reactive epitopes between SVV and VZV [6–8], it might be enough to show only the seropositivity of VZV IgG in rhesus monkeys. Although there were no comparators, such as a subcutaneously administered group and a intratracheal inoculation administered group, at the group-setting stage, our vaccination strategy produced a strong positive VZV IgG response in all vaccinated animals and suggests further testing. This is because the seropositivity of VZV IgG had to be achieved in a short time to bring the monkey into our facility where the SVV outbreak occurred.

We used 0.7 ml of SuduVax as an intratracheal inoculation. This volume might be negligible in terms of safety concerns. A typical volume of 1–2 mL/kg body weight was safely used to avoid any potential coughing (> 2 ml/kg) and subsequent loss of instilled test material [9].

## Conclusions

Our results showed that clinically available SuduVax was able to induce VZV IgG production, especially after two intratracheal inoculations at one-week intervals. These data show a strong humoral immune response was produced by VZV vaccination via intratracheal inoculation. They suggest further efficacy testing against SVV challenge is needed to confirm the use of VZV vaccination to protect against SVV reactivation and disease in rhesus monkeys utilized for immunosuppressive and organ transplantation studies.

## Methods

All animal experiments were approved by the Institutional Animal Care and Use Committee (IACUC) of the Biomedical Research Institute at the Seoul National University Hospital, an AAALAC accredited facility (IACUC approval number: 15–0297-C1A0).

**Fig. 4:**
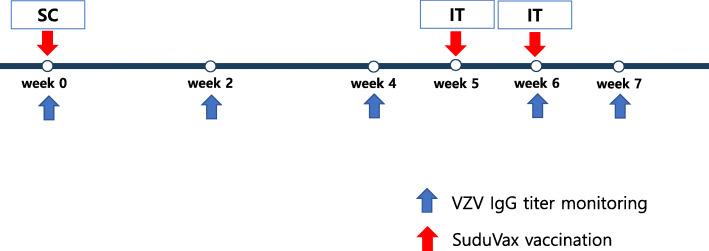
Time schedule for SuduVax vaccination and VZV IgG titer monitoring. SC; subcutaneous injection. IT; intratracheal inoculation

After the SVV outbreak and follow-up in our NHP facility, we planned to immunize newly imported macaques using clinically available human VZV vaccine. SuduVax (VZV MAV strain; a live attenuated form, Green Cross, Yongin, Korea) was used to immunize rhesus monkeys. Sixteen naïve female rhesus monkeys (age: 3–5 years) were used. Sedation of the monkeys was induced with medetomidine (0.2 mg/kg, IV) and ketamine (5 mg/kg, IV). The injection site was prepared aseptically. A vial of the vaccine solution (0.7 ml, 1400 pfu) was subcutaneously injected at week 0 and administered via intratracheal inoculation at week 5 and 6 due to poor VZV IgG seropositive after 0 week subcutaneous VZV inoculation. The trachea of the rhesus monkey was grasped by the thumb and index finger of the nondominant hand. The needle was inserted into the trachea in a 30-degree cephalad or caudal orientation to the skin and directed cranially or caudally after penetration of the anterior tracheal wall. Before inoculation, air was aspirated to ensure that the needle was in the proper position. The complications related to subcutaneous or intratracheal injection were monitored by observer’s subjective judgement for pain (hand touching on the injection sites, the degree of coughing and screaming, decreased activity, and reduced food intake) and observer’s objective judgement for swelling and redness at the injection site, fever, and rash by systemic reaction. VZV IgG titers were monitored at week 0, 2, 4, 6, and 7 (Fig. 4).

Two milliliters of blood was collected from the left saphenous vein on weeks 0, 2, 4, 6, and 7. VZV IgG titers were measured by chemiluminescent immunoassay (Liaison™ VZV IgG, DiaSorin S.p.A., Italy). VZV IgG titers greater than 150 mIU/ml were considered seropositive VZV IgG titers greater than 150 mIU/ml were considered seropositive which is suggested by chemiluminescent immunoassay (Liaison™ VZV IgG, DiaSorin S.p.A., Italy) for human VZV [10], cutoff value for rhesus monkey is not existent because VZV is not the rhesus monkey’s disease.

To determine how the VZV IgG antibody titer waned over time, we analyzed five monkeys among 16 at one of three time points among 19, 23, 31, and 55 weeks.

## Data Availability

Not applicable.

## Abbreviations

AAALAC: Association for Assessment and Accreditation of Laboratory Animal Care
IACUC: Institutional Animal Care and Use Committee
IT: Intratracheal inoculation
SC: Subcutaneous injection
SVV: Simian varicella virus
VZV: Varicella-zoster virus
